# Adsorbate-driven cooling of carbene-based molecular junctions

**DOI:** 10.3762/bjnano.8.206

**Published:** 2017-10-02

**Authors:** Giuseppe Foti, Héctor Vázquez

**Affiliations:** 1 Institute of Physics, Academy of Sciences of the Czech Republic, Cukrovarnicka 10, Prague, Czech Republic

**Keywords:** adsorbate, carbene, current-induced heating and cooling, molecular junction, vibrations

## Abstract

We study the role of an NH_2_ adsorbate on the current-induced heating and cooling of a neighboring carbene-based molecular circuit. We use first-principles methods of inelastic tunneling transport based on density functional theory and non-equilibrium Green’s functions to calculate the rates of emission and absorbtion of vibrations by tunneling electrons, the population of vibrational modes and the energy stored in them. We find that the charge rearrangement resulting from the adsorbate gates the carbene electronic structure and reduces the density of carbene states near the Fermi level as a function of bias. These effects result in the cooling of carbene modes at all voltages compared to the “clean” carbene-based junction. We also find that the direct influence of adsorbate states is significantly smaller and tends to heat adsorbate vibrations. Our results highlight the important role of molecular adsorbates not only on the electronic and elastic transport properties but also on the current-induced energy exchange and stability under bias of single-molecule circuits.

## Introduction

Molecular electronics has experienced a remarkable progress since its first proposal [1]. Theoretical as well as experimental advances have made it possible to achieve a detailed understanding of the main factors governing single-molecule transport [2–4]. Recently, energy-exchange processes between tunneling electrons and vibrational degrees of freedom have been considered. Understanding heat generation and dissipation in the molecular junction is particularly relevant for the stability of molecular circuits under bias [5–10]. However, simulations of single-molecule studies have usually assumed rather idealized conditions with “clean” junctions in which no molecular species other than the conducting molecules are considered. STM break-junction measurements are often carried out in solution, where, in addition to target molecules, solvent molecules are also present [11–16]. The presence of contaminants that might diffuse on the surface and cause fluctuations in conductance or sudden changes in the junction stability cannot be ruled out [17]. Finally some molecular end groups are cleaved at the junction [18–22]. These chemical species presumably remain in the vicinity of the junction although their role is difficult to analyze precisely, because single-molecule break-junction experiments provide only indirect information on the nature of the junction from conductance and forces [14,23–24]. In spite of this, the role of molecular co-adsorbates in elastic and inelastic transport processes has not been studied in detail. Therefore going beyond the approximation of a “clean” junction and analyzing the effect of adsorbates is particularly relevant.

Here we address this issue by investigating the effect on the current-induced heat exchange of a molecular species adsorbed in the vicinity of the molecular junction. As we show in this paper, the presence of adsorbates not bonded directly to the molecule gives rise to pronounced deviations from its behavior as an isolated molecule, resulting in marked changes in the heating and cooling dynamics (HCD) of the junction.

We consider a N-heterocyclic carbene-based junction and study the effect of a neighboring NH_2_ species adsorbed on one of the electrodes, close to the metal–carbene bond. N-Heterocyclic carbenes (NHCs) have recently attracted much attention because of their interesting electronic properties and their high thermal and mechanical stability [25–27]. We recently studied the electronic and elastic transport properties of NHC-based junctions anchored to Au(100) electrodes [28]. We reported a strong dependence of transport properties on the atomistic structure of the metal/molecule interface and analyzed its implications on the current-induced damping and excitation of localized molecular vibrations [29]. We considered the case of a “clean” junction formed by the NHC only. Here, in turn, we extend this study and compare the current-induced heating of vibrational modes for two junctions: i) a “clean” metal/NHC/metal junction and ii) an asymmetric metal + NH_2_/NHC/metal junction where a NH_2_ group is adsorbed on one of the leads. We use a first-principles, self-consistent description of the junction out of equilibrium based on density functional theory (DFT) and non-equilibrium Green’s functions (NEGF). We show how the change in the electronic structure of the junction induced by the presence of the adsorbate promotes the cooling of NHC vibrational modes through i) electrostatic gating of molecular levels and ii) quenching of carbene density of states (DOS) as a function of the applied bias. We illustrate the connection between the gating of NHC states and the heating of the junction by comparing vibron populations as a function of bias for two systems: the “clean” NHC structure and a modification of this system where the spectrum was shifted so that the LUMO position matches that of the geometry with NH_2_. Based on this analysis, we are able to clearly discriminate the contribution to the cooling of carbene modes of the gating from that of the reduction of carbene DOS near the Fermi level due to the adsorbate. The current-induced heating of adsorbate modes reveals the important role of molecule–adsorbate through-space tunneling. By setting the adsorbate electronic structure elements to zero in the calculations we could also quantify the direct role of NH_2_ states in vibron populations. We establish a connection between this change and the degree of localization of vibrational modes on the adsorbate. This work shows that adsorbate species in the vicinity of the junction can influence the current-induced heating and cooling of vibrational modes of the conducting molecule.

## Geometries and Computational Details

**Figure 1:**
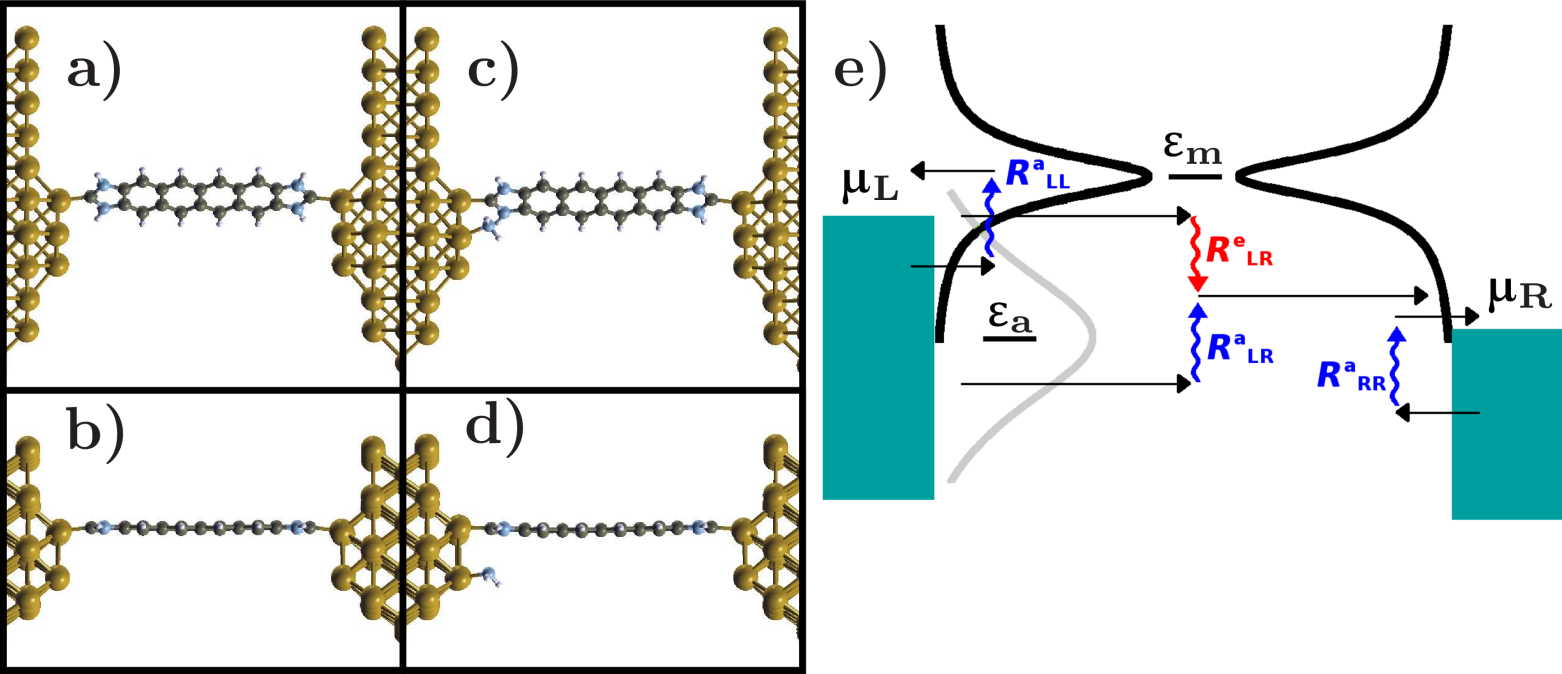
a,b) Front and side view of the NHC attached to clean electrode terminations (**C** structure). c,d) Front and side view of the junction in presence of a NH_2_ group adsorbed on one side of the junction (**CA** structure). e) Schematic illustration of the different emission and absorption processes due to tunneling electrons. The energy ε_m_ represents the state associated to the molecule bridging the two electrodes while ε_a_ is the state of an adsorbate attached to the left electrode. The black curves are the left- and right-projected DOS of the molecule while the gray curve is the density of states of the adsorbate.

Figure 1a–d shows the structures considered in the present study. The conducting unit is a NHC-based molecule formed by four benzene rings and two imidazole groups as linkers attached to Au(100) electrodes with tetrameric tip terminations (structure **C**). We compare this to a junction (structure **CA**) having the same NHC-based molecule with a NH_2_ group adsorbed on one of the tetramers, close to the Au–carbene bond but not chemically bound to the carbene. We consider the junction with the NH_2_ unit adsorbed at one of the electrodes only so as to introduce asymmetries in the junction DOS under bias, given that NH_2_ states will be pinned to the left chemical potential.

We use Siesta [30] and TranSiesta [31] for the structure relaxation and the calculation of the electronic and transport properties. We use a single-ζ plus polarization basis for gold and a double-ζ plus polarization basis for nitrogen, hydrogen and carbon atoms. Exchange–correlation is described with the generalized gradient approximation (GGA) [32]. For the calculation of the electron–phonon coupling matrix we use the Inelastica code [33] with the **M**(Γ) approximation [34], which consists of calculating the electron–phonon coupling matrix **M** in just one point of the Brillouin zone (Γ) for both electrons and phonons. The dynamical region includes the molecule, the Au atoms forming electrode terminations and the adsorbate atoms.

The position of the molecule, tip atoms and the surface gold layers were relaxed until residual forces fell below 0.02 eV/Å. We used a *k* = 5 × 5 Monkhorst–Pack grid for the calculation of the electronic structure. Eigenchannels are calculated as described in [35]. The electronic structure of the junction is calculated self-consistently in presence of an external bias *V*_b_ ranging from 0.2 to 1.2 V in steps of 0.2 V.

The current flowing through the junction drives the vibronic system out of equilibrium, heating the vibrational modes of the molecule. The different processes exchanging energy between electronic and vibrational degrees of freedom are shown in Figure 1e. The energies ε_m_ and ε_a_ represent the molecular and adsorbate states, respectively. The adsorbate state is pinned to the left chemical potential μ_L_. Black curves represent the left- and right-projected molecular DOS while the gray curve is the DOS of the adsorbate.


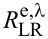
 is the emission rate of vibrational quanta of mode λ due to tunneling electrons. Electrons flowing through the junction from the left electrode can lose energy by scattering inelastically with molecular vibrations and tunnel into the right electrode at a lower energy. Since this transfers energy to vibrations localized at the interface, these processes result in the heating of the molecule. Electrons can also absorb energy from the vibronic system through intra- or inter-electrode inelastic scattering processes (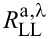, 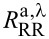 and 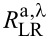 in Figure 1e). These mechanisms transfer energy from molecular vibrations to tunneling electrons and thus contribute to the cooling of the junction. They add up to a total rate *R*^a^*^,λ^*. These emission and absorption processes have been described in detail elsewhere [5–6,29,36]. Finally, vibrations can also dissipate energy into bulk phonons.

The steady state solution for the population of vibrational mode λ (excluding anharmonic coupling between vibrational modes) is [36–38]:

[1]
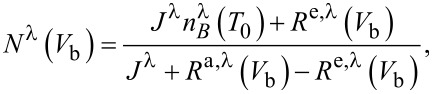


where the coupling of vibrations to tunneling electrons and to bulk phonons are both taken into account. The parameter 
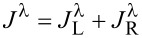
 describes the damping of molecular vibrations with bulk phonons in the left (L) and right (R) contacts and tends to drive the system to the Bose–Einstein (BE) distribution 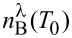 of the bath at a temperature *T*_0_ (see below). For *J*^λ^ = 0, energy cannot be dissipated into bulk phonons and the junction is in the undamped limit [29]. On the other hand, for large *J*^λ^, coupling to Au phonons is strong and current-induced excitations are completely damped by bulk phonons. In the intermediate limit that we consider here, the current can excite the vibrational system beyond the dynamics dictated by Au phonons.

The processes of emission and absorption of molecular vibrations due to the interaction with the electronic system has been derived in the framework of NEGF theory [6,37,39]. In the low temperature limit the emission rate 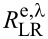 of vibrational quanta by tunneling electrons is expressed as:

[2]
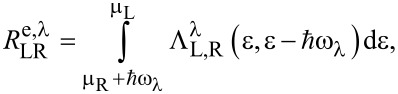


while the absorption rates are given by:

[3]
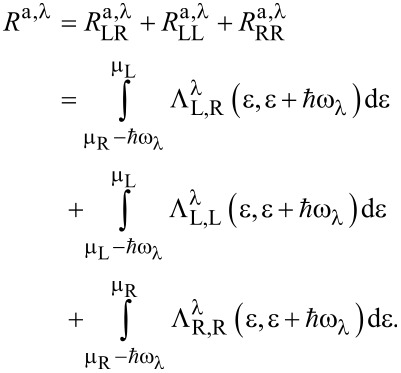


The matrices 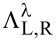 are given by the product of the electron–vibration couplings and the left and right spectral densities:

[4]
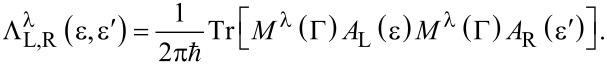


In our analysis the damping term *J*^λ^ in Equation 1 is introduced as an external parameter. We assume a constant damping of vibrational modes into bulk phonons of *J*^λ^/2 = *J*_L_ = *J*_R_ = 5 × 10^10^ Hz. In the following we thus omit the label λ in the notation for the parameter *J*. This value of *J* falls in the range of previous ab initio calculations [37,40] and ensures at the same time a relatively low effective temperature [40] of the junction even at high bias (1.2 V). A stronger damping parameter would dominate over the electronic contributions driving the system to the BE distribution. We also assume a relatively low temperature of the bath *T*_0_ of 100 K. Changing the temperature of the bath would roughly shift the offset of accumulated energy of the vibrational modes and of the effective temperature of the molecule [37].

## Results and Discussion

### Effect of the NH_2_ adsorbate on the electronic structure of the clean junction

We first analyze the effect on the electronic structure of an NH_2_ unit adsorbed in the vicinity of the NHC molecule on one side of the junction. We consider the spectral properties of a “clean” junction (labeled **C**) consisting of the NHC only. This is shown in Figure 1a,b. We compare this to a junction called **CA** which, in addition to the NHC, has an NH_2_ adsorbate on one of the electrode terminations (Figure 1c,d).

**Figure 2:**
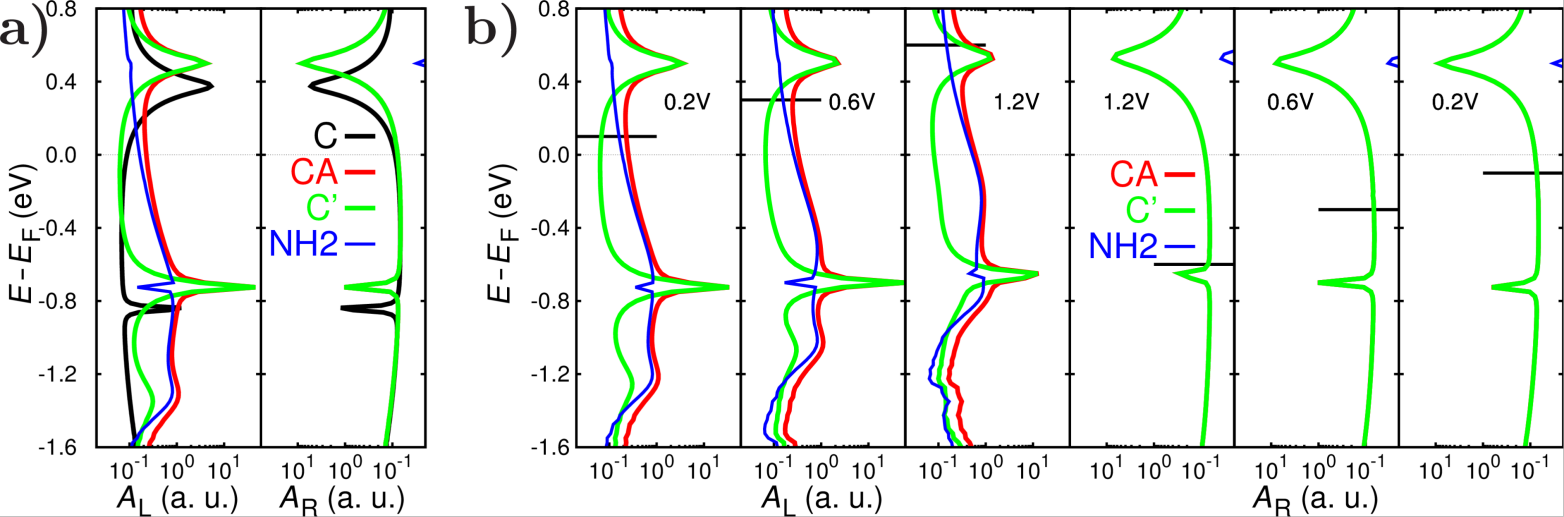
a) Left and right spectral functions at 0 V for a clean NHC (**C**, structure shown in Figure 1a,b) and with a NH_2_ group adsorbed on the left electrode, (**CA**, structure in Figure 1c,d). For the latter we decompose the contributions of the carbene backbone and adsorbate (**CA = C’ + NH_2_**); b) Left (left panels) and right (right panels) projected spectral functions of the **CA** junction decomposed into the contributions from the NHC backbone and from the NH_2_ adsorbate at 0.2, 0.6 and 1.2 V. Black, horizontal lines represent the left and right chemical potentials at different biases. NH_2_ states move with respect to NHC states under bias and, at 1.2 V, there is a significant NH_2_-derived DOS above the HOMO.

Figure 2a shows the left- and right-projected spectral functions (*A*_L_(ε) and *A*_R_(ε), respectively) for the two junctions **C** and **CA** at equilibrium (no bias applied). The spectral functions are related to the DOS of the left and right metal/molecule interfaces [41]. The presence of the adsorbate on one side of the junction induces an up-shift of approximately 0.13 eV of the NHC levels. Also, below the Fermi level the adsorbate introduces broad features to *A*_L_(ε). On the right side of the junction no NH_2_ states are visible in *A*_R_. Importantly, at high applied voltages, a significant part of NH_2_ DOS is almost completely above the HOMO of NHC (Figure 2b). Thus the adsorbate introduces an asymmetry in the electronic structure of the **CA** junction.

Electron donation from the carbene to the Au tip is reduced in the presence of the NH_2_ unit, resulting in NHC spectral features at slightly higher energies in the **C** than in the **CA** junctions. Notice that the behavior in terms of charge transfer and redistribution of this NH_2_ adsorbate is fundamentally different from that of anchoring groups in molecular junctions (R–NH_2_): here NH_2_ acts as an acceptor, while as anchoring group, amines are electron donors [42]. In Supporting Information File 1, we show a comparison of the plane-averaged electron density difference upon adsorption of NH_2_ for reference, and its saturated counterpart NH_3_ (which is similar to the role as anchoring group R–NH_2_). Similar shifts of molecular levels are expected for other adsorbates, whose donor or acceptor character could be used to gate NHC conducting orbitals.

**Figure 3:**
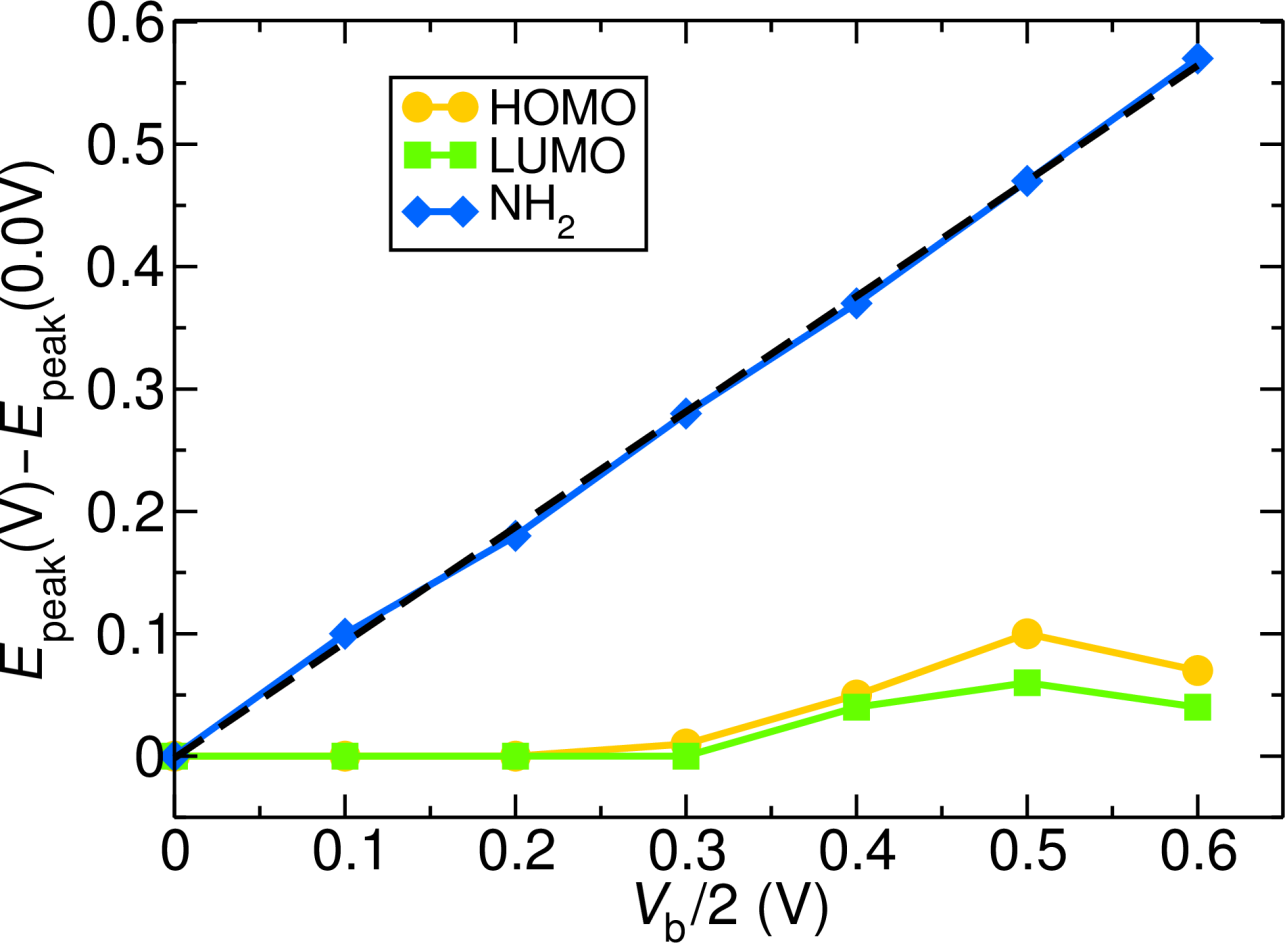
Shift of HOMO, LUMO and NH_2_ states with respect their equilibrium position as a function of the applied bias *V*_b_ in the **CA** junction. The black dashed line represents a linear regression fit with slope of 0.942. Adsorbate states follow the electrostatic potential of the left electrode. NHC states are not pinned to either electrode and exhibit a non-monotonic behavior with applied voltage, as discussed in the text.

We now turn to the spectral features of the **CA** junction as a function of applied voltage. Figure 2b shows the left and right projected spectral functions *A*_L_(ε) and *A*_R_(ε) decomposed into NHC (**C’**, green) and adsorbate (NH_2_, blue) components for three different values of the external bias *V*_b_: 0.2, 0.6 and 1.2 V. Carbene and NH_2_ states have a different bias dependence. While adsorbate states are strongly pinned to the chemical potential of the left electrode, NHC states do not clearly follow the potential of either electrode. Figure 3 shows the pinning of the molecular HOMO and LUMO and of the states of the adsorbate.

The figure tracks the shift in the peak positions with respect to the value at equilibrium (0 V). Up to 0.6 V the positions of HOMO and LUMO are essentially unchanged. After this they move up with applied bias but then down after *V*_b_ = 1 V. This behavior can be explained by noting that half the voltage is applied to each electrode. With increasing bias, the left chemical potential approaches the LUMO peak while the right one approaches HOMO. At *V*_b_/2 = 0.3 V the left chemical potential approaches the LUMO and NHC features move up to avoid charging. At *V*_b_/2 = 0.5 V, however, the right chemical potential is close to HOMO and there is a balance between the filling of the LUMO resonance by the left electrode and the emptying of the HOMO by the right lead. The drop in NHC spectral features in Figure 3 beyond 0.5 V shows that the latter process dominates. In contrast to NHC features, the NH_2_-derived peak in the DOS is strongly pinned to the left electrode and moves up linearly with applied voltage.

### Population and energy of vibrational modes

**Figure 4:**
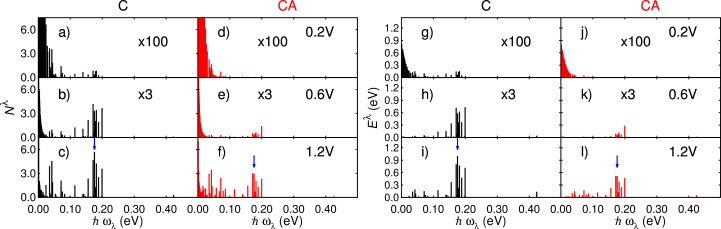
a–f) Populations of all vibrational modes at 0.2, 0.6 and 1.2 V for the junction with (CA) and without (C) the NH_2_ adsorbate. g–l) Average energy of all modes for the two structures considered at 0.2, 0.6 and 1.2 V. Blue arrows indicate the mode at 175 meV studied in detail in the text.

In the presence of an external bias the vibronic system is driven out of equilibrium by the electrical current. Figure 4a–f shows the populations *N*^λ^(*V*_b_) of each vibrational mode at 0.2, 0.6 and 1.2 V for the two junctions considered: the “clean” junction **C** and the junction with the NH_2_ adsorbate in the vicinity, **CA**. Two distinct regions can be clearly seen, consistent with the character of the junction vibrational modes: At energies higher than ca. 100 meV, vibrational modes are characterized by in-plane movement of atoms forming the NHC backbone. Out-of plane NHC modes are present mostly below about 100 meV. NH_2_ modes are found throughout the spectrum up to ca. 420 meV (Figure S3, Supporting Information File 1).

At low bias (0.2 V) the population of vibrational modes follows a Bose–Einstein distribution for **C** and **CA**, with low-energy modes populated (Figure 4a,d). At 0.6 V higher-energy modes of the **C** junction get populated while the mode populations for the **CA** junction roughly still follow a BE distribution (Figure 4b,e). At 1.2 V the electronic current has established a clearly non-equilibrium vibronic distribution for both junctions (Figure 4c,f).

These changes are reflected in the energy stored in vibrational modes, which gives a measure of the heating as a function of the applied bias. From Equation 1 the energy stored in each mode can be calculated as 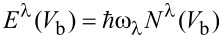. Figure 4g–l shows this quantity. At 0.2 V very little energy is stored in vibrational modes across the spectrum. At higher voltages, substantial energy is stored in modes at below ca. 200 meV, which correspond to vibrations of the NHC backbone. Vibrational modes localized on the adsorbate are found at 58, 86 and 407 meV. Importantly, even if the NH_2_ group is not directly attached to the right electrode and not chemically bound to the NHC, the stored energy of these three modes increases as a function of the applied bias. This finding emphasizes the important role of through-space tunneling supported by the molecular backbone bridging the two electrodes in the HCD of the adsorbate. In Supporting Information File 1 we show the populations and stored energy of all modes calculated using a smaller damping parameter *J* = 4 × 10^10^ Hz.

### Connection between DOS and heating of NHC modes

The populations and average energies of NHC modes in the presence of the NH_2_ adsorbate (junction **CA**) are lower than for the junction without the adsorbate (**C**). This can be rationalized in terms of changes in the electronic structure of the NHC backbone induced by the presence of the NH_2_ group: i) an up-shift of carbene features and ii) a pronounced decrease of the LUMO peak of the left spectral function with bias.

First, the up-shift of NHC states for the **CA** structure results in a reduction of carbene DOS in the integration window near the Fermi level (black and green curves in Figure 2a). As we discuss later, in the **CA** junction NH_2_ states do not appreciably affect the energy exchange dynamics of carbene modes, which are dominated by carbene DOS. Thus the reduction of carbene DOS near the Fermi level compared to the **C** structure results in smaller emission rates 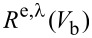 in Equation 1.

Second, the LUMO peak in the *A*_L_(ε) becomes lower as a function of applied voltage (Figure S5 in Supporting Information File 1 shows the left-projected spectral function at 1.0 V for the two structures). This reduction is observable for both the **C** and **CA** structures but it is more pronounced in the presence of the adsorbate. This effect also contributes to the decrease of emission rates *R*^e^*^,λ^*(*V*_b_). Thus, at any given bias, the emission rate of a vibrational mode λ will be lower for the structure with the NH_2_ adsorbate.

We now discuss the connection between vibron populations, the emission rate *R*^e^*^,λ^*(*V*_b_) and the DOS structure by analyzing the terms in Equation 1. We find that, for high energy modes (*E* ≥ 70 meV), the numerator can be well approximated by the emission rate *R*^e^*^,λ^*(*V*_b_) since, assuming a low phonon-bath temperature, 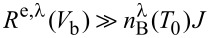. Also, the denominator *J* + *R*^a^*^,λ^*(*V*_b_) − *R*^e^*^,λ^*(*V*_b_) does not change much for carbene modes over the whole bias range considered. Figure S6 in Supporting Information File 1 shows the bias dependence of the denominator in Equation 1 for a couple of modes at low (35 meV) and high (176 meV) energy for the **C** structure. These oscillations around *J* as a function of *V*_b_ are relatively small as long as molecular resonances are broad compared to the mode energy 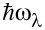 (a condition generally met for carbene–metal junctions, with the exception of long, chain-like structures [29]). Thus we find that, for carbene modes, Equation 1 can be approximated as follows:

[5]
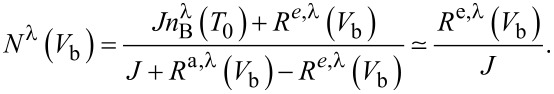


Since the emission rate *R*^e^*^,λ^*(*V*_b_) depends directly on the DOS inside the Fermi window, the previous approximation provides a more intuitive understanding of the connection between vibron occupation *N*^λ^ and the electronic structure. A higher DOS results in a higher emission rate and thus a higher occupation number while a reduction of the DOS in the bias window would result into a lower *R*^e^*^,λ^*(*V*_b_) and, consequently, a lower *N*^λ^. We now illustrate the validity of Equation 5 by focusing on the stored energy of a representative mode. The mode at 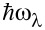 = 176 meV is delocalized over the carbene backbone (see Figure S7, Supporting Information File 1) and has one of the highest populations and stored energy under bias. It is highlighted in Figure 4 with blue arrows.

**Figure 5:**
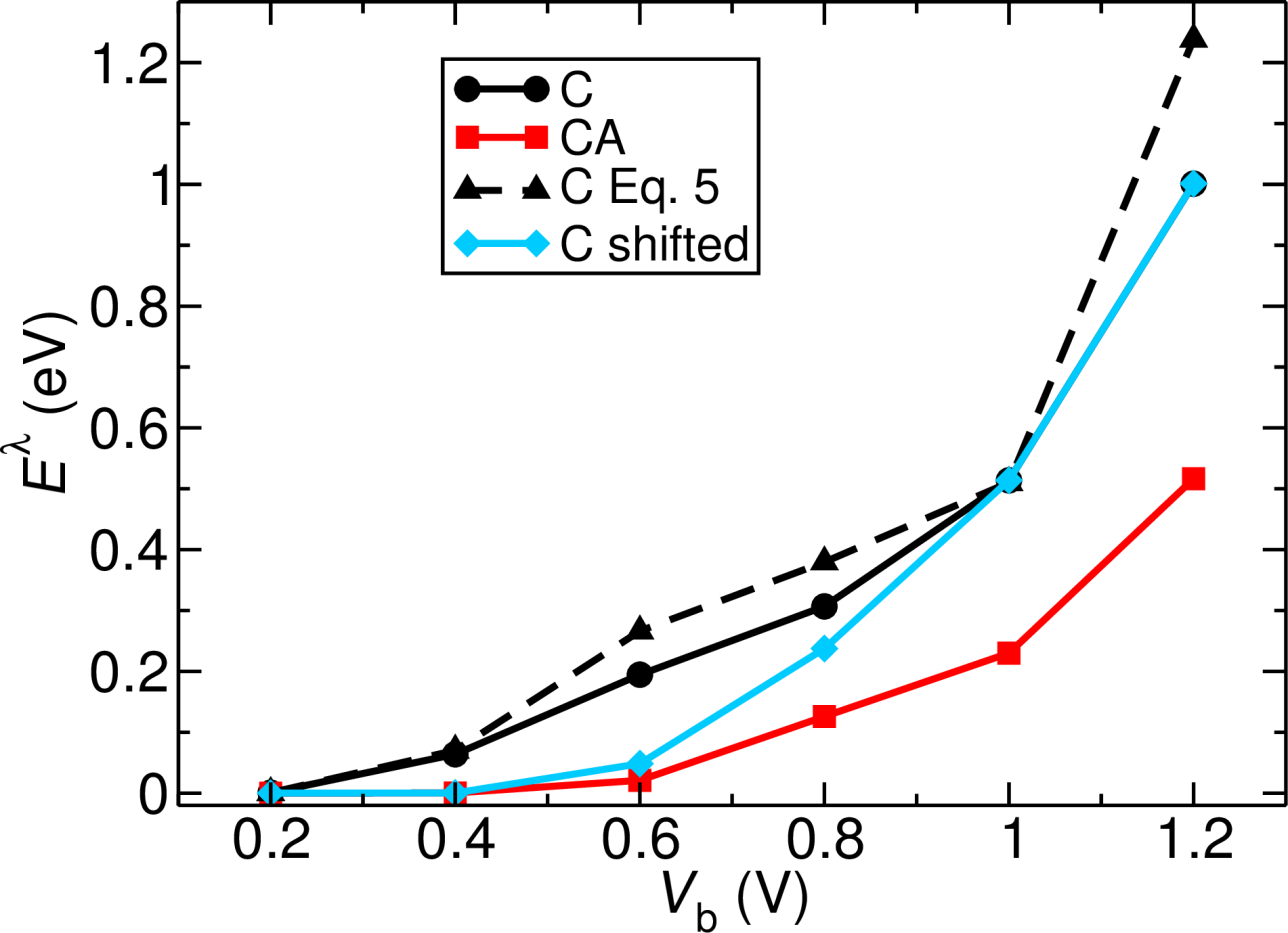
Energy stored in the mode at 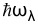 = 176 meV as a function of applied bias for the **C** and **CA** junctions, and for a **C** junction with spectral functions shifted so as to match the LUMO of the **CA** structure. The black, dashed line represents the stored energy for structure **C** calculated using Equation 5.

The two black curves in Figure 5 represent the energy stored in this vibrational mode for the **C** structure calculated according to Equation 1 (continuous line) and Equation 5 (dashed line), respectively. In the bias regime below 1.2 V, Equation 5 provides a good approximation to the computationally more expensive calculation needed for vibron population using Equation 1.

For this mode we now quantitatively evaluate the effect on the HCD of the gating induced by the NH_2_ adsorbate by considering the **C** structure and shifting its spectrum such that the LUMO position matches that of the **CA** junction. This ensures that other effects introduced by the adsorbate (e.g., hybridization) are excluded. We note that the pinning behavior of the LUMO in the **C** and **CA** junctions is slightly different and therefore the LUMO peaks are matched at each applied voltage. Figure 5 shows the calculated energy stored in this vibrational mode also for the **CA** and shifted **C** structures. The energy of the vibrational mode grows as a function of the applied bias and is clearly higher for the **C** junction over the whole bias range. The blue curve shows how, in the low bias regime, an up-shift of NHC states results in a relatively strong reduction of vibron energy. The energy stored in the **CA** junction can be replicated by shifting the **C** LUMO. Above 0.8 V, however, the LUMO position in the **C** and **CA** structures approximately coincide and the applied shift is negligible. In this voltage range the main difference between both structures is no longer the position but the height and width of the LUMO. This result demonstrates how, for systems with LUMO-dominated transport, a rigid up-shift of molecular levels arising from, e.g., adsorbate-induced chemical doping can promote the cooling of the molecular junction in the low bias regime.

### HCD mediated by adsorbate states

As previously mentioned, the NH_2_ adsorbate introduces additional features in the low-energy **CA** DOS (blue line in Figure 2a). The position and bias dependence of these states can influence the heating and cooling dynamics of the junction. To quantitatively evaluate this effect for the **CA** junction we calculate vibron populations setting the matrix elements of the left and right spectral functions involving atomic orbitals of the adsorbate to zero in Equation 2 and Equation 3. We compare these results to the calculation that includes all contributions. We checked that setting the elements of the electron–vibration coupling matrix M^λ^ to zero would yield the same result. This is expected since the energy-dependent rates 
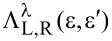
 are obtained from the trace of the matrix products (Equation 4) where only diagonal terms contribute. Notice also that a direct comparison between the **C** and **CA** structures would not be meaningful since modes and mode energies change, and carbene and adsorbate states are hybridized.

We derive the change in the accumulated energy of each mode using the following expression:

[6]
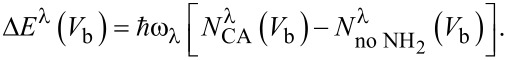


Here 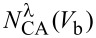 is the population of vibrational mode λ including all contributions of the spectral functions, while 
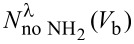
 is the one obtained by setting the contributions involving adsorbate orbitals to zero, as described above. A positive value of Δ*E*^λ^(*V*_b_) means that the adsorbate DOS tends to increase the accumulated energy of a vibrational mode λ. A negative value, on the other hand, means that adsorbate states contribute to cool down a certain vibrational mode. From Equation 6 on can see that a given change in vibron population has a stronger impact on high energy modes because of the prefactor 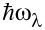.

Much insight can be gained by correlating the character and localization of each mode with the energy it stores. For this we calculated how much each vibrational mode involves the NH_2_ adsorbate using the following projection:

[7]
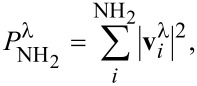


where 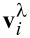 are the amplitudes of eigenmode λ and the sum extends over the NH_2_ group.

**Figure 6:**
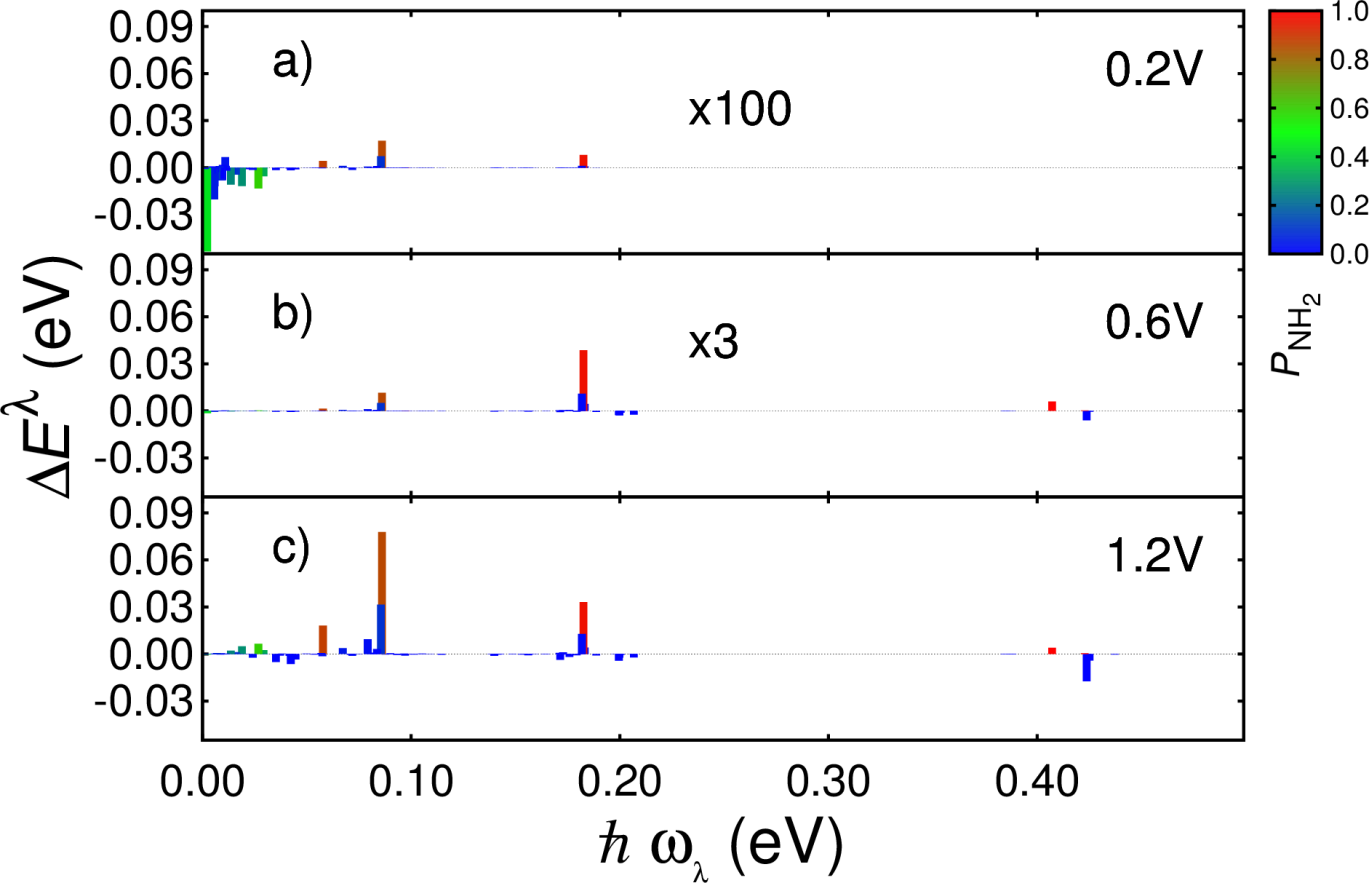
Change in energy stored in each vibrational mode when setting the contributions of the NH_2_ group to zero in the spectral functions of the **CA** junction at (a) 0.2 V, (b) 0.6 V and (c) 1.2 V for *J* = 1 × 10^11^ Hz. The color bar indicates to which extent the vibrational modes are localized on the adsorbate, as given by Equation 7.

Figure 6 shows the difference between the energies of the vibrational modes of the **CA** junction and with the contributions of the NH_2_ group switched off. In Supporting Information File 1 we show the same calculation using a smaller damping parameter *J* = 4 × 10^10^ Hz. The color code represents the projection of each mode on the NH_2_ adsorbate [Equation 7]. Red color indicates modes with a strong weight on the adsorbate while modes mostly localized on the carbene backbone are identified by the blue color. Comparing the energy scales of Figure 6 to that of Figure 4 and Figure 5, we see that the overall change in the energy stored in vibrational modes induced by adsorbate states is small compared to the values for the **C** and **CA** junctions, and small compared to the changes in *E*^λ^(*V*_b_) resulting from the LUMO shifts.

Figure 6 shows how the changes Δ*E*^λ^ are negligible at low bias (0.2 V). At 0.6 V the most significant change is a modest heating of a mode at 182 meV, which is mostly localized on the NH_2_ group. At 1.2 V the change in average vibron energy becomes higher, also for two additional modes at 58 meV and 86 meV, both of which have large amplitudes 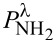 on the adsorbate. The strong decrease for the mode at 423 meV (localized on carbene backbone) is mainly due to the large prefactor 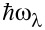 (Equation 6). Calculations for a smaller value of *J* (Figure S8, Supporting Information File 1) show how the impact of adsorbate DOS becomes more important with weak damping by bulk phonons. In particular for high-energy modes, even a relatively small change in vibron population results in an appreciable change of the average energy of the mode, such as the NHC mode at 423 meV.

Thus the heating of NHC modes is essentially unaffected by the NH_2_ DOS, and the electronic structure of the adsorbate has little direct influence on the heating of carbene modes. Moreover the adsorbate DOS tends to heat mostly NH_2_-localized modes. This analysis illustrates the heating of adsorbate modes via through-space tunneling and quantifies the role of adsorbate DOS in this process.

## Conclusion

We studied the effect of a molecular adsorbate on the current-induced heating and cooling dynamics of a NHC-based molecular junction. We calculated the bias-dependent rates of emission and absorption of molecular vibrations using first principles methods based on DFT-NEGF. We considered an electron-withdrawing NH_2_ species adsorbed in the vicinity of the molecule on one of the electrodes and found that it cools carbene modes with respect to the junction with no adsorbate. The main result is that the influence of the adsorbate on the cooling of carbene modes is indirect, resulting from the changes in the electronic structure of carbene brought by the presence of the adsorbate rather than from the electronic structure of the adsorbate itself. Introduction of the NH_2_ species shifts NHC states up and reduces the height of the LUMO peak with respect to the junction without the adsorbate. These effects lead to a reduction of the DOS in the integration window in the calculation of the emission rate *R*^e^*^,λ^*. Thus the population of carbene modes and the energy stored in them decreases with the adsorbate in the vicinity, and carbene modes are effectively cooled under bias by the presence of the NH_2_ species. Adsorbate states are present as broad features below the Fermi level in the junction DOS. The impact of this DOS in the dynamics of energy exchange is not as large as through the modification of the carbene DOS described above. Adsorbate states tend to heat modes localized on the adsorbate. This results from through-space tunneling since adsorbate and carbene are not bonded to each other. In summary, we have shown that a species adsorbed in the vicinity of a molecular junction can influence the heating and cooling dynamics of the conducting molecule. This affects not only the elastic transport properties but also the stability of molecular junctions under an applied bias. We therefore expect this work to be of general interest in the broader field of single molecule transport.

## Supporting Information

File 1 The Supporting Information contains the following material: electron density difference upon adsorption of NHC over a clean and a NH_2_-decorated surface; electron density difference upon adsorption of NH_2_ and NH_3_; calculated populations and stored energies of all vibrational modes using a smaller damping parameter *J*; all emission and absorption rates as a function of bias for two modes (35 meV and 176 meV) for the **C** structure; change in the stored energy associated to the adsorbate DOS; a picture of the vibrational mode at 176 meV for the **CA** structure.
