# Supplementary material for: Adsorbate-driven cooling of carbene-based molecular junctions
Source: Beilstein J Nanotechnol. 2017 Oct 2;8:2060–8. doi: 10.3762/bjnano.8.206 (PMC5647705; doi:10.3762/bjnano.8.206)
Supplement: File 1 — The Supporting Information contains the following material: electron density difference upon adsorption of NHC over a clean and a NH2-decorated surface; electron density difference upon adsorption of NH2 and NH3; calculated populations and stored energies of all vibrational modes using a smaller damping parameter J; all emission and absorption rates as a function of bias for two modes (35 meV and 176 meV) for the C structure; change in the stored energy associated to the adsorbate DOS; a picture of the vibrational mode at 176 meV for the CA structure. [file Beilstein_J_Nanotechnol-08-2060-s001.pdf]

Supporting Information

for

Adsorbate-driven cooling of carbene-based molecular junctions

Giuseppe Foti* and Héctor Vázquez*

Address: Institute of Physics, Academy of Sciences of the Czech Republic, Cukrovarnicka 10, Prague, Czech Republic

Email: Giuseppe Foti - foti@fzu.cz, Héctor Vázquez - email vazquez@fzu.cz

* Corresponding author

Additional computational data

Electron density difference upon adsorption of carbene

Figure S1 shows the electron density difference upon adsorption of carbene over a) the clean and b) decorated electrode. The presence of the fragment perturbs the charge redistribution at the metal/molecule interface, resulting in a smaller charge depletion at the Au-C bond. As a consequence, the induced electric field (Figure S0c) on the side of the adsorbate is slightly lower compared to the case of clean electrode. The associated (negative) potential energy shift is thus lower when the NH_2 fragment is adsorbed in close proximity to the junction (Figure S1d) and Carbene orbitals are at a slightly higher energy with respect to the case of adsorption over the clean electrode. From Figure S1b the adsorption of carbene induces an accumulation of electrons on the NH_2 group which thus acts as electron acceptor. This down-shift of molecular levels is expected to be stronger for stronger charge acceptors.

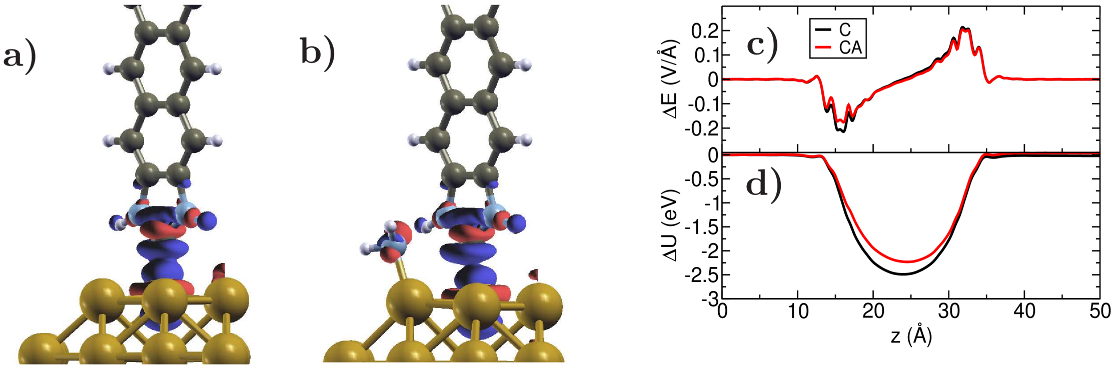

Figure S1: Electron density difference associated to the adsorption of Carbene on a) the clean electrode surface and b) in presence of the NH_2 fragment. The presence of the NH_2 fragment inhibits charge depletion at the Au-C bond. c) Electric field induced by the charge redistribution across the junction with and without the NH_2 fragment. On the left side of the junction the maximum of the induced electric field is lower for the geometry with the decorated electrode. d) Potential energy shift induced by the metal-molecule coupling with and without the NH_2 fragment.

The shift of HOMO and LUMO, in the presence of the NH_2 fragment can be easily explained in terms of the interface dipole generated by the chemical bond between Carbene and Au electrodes.

We thus calculate the charge redistribution associated to this bond:

$$\overline{\rho}_{\text{diff}}(z) = \overline{\rho}_{\text{jcn}}(z) - [\overline{\rho}_{\text{mol}}(z) + \overline{\rho}_{\text{metal}}(z)], \quad (1)$$

where $\overline{\rho}_{\text{jcn}}(z)$ is the electron density of the metal/molecule/metal junction while $\overline{\rho}_{\text{mol}}(z)$ and $\overline{\rho}_{\text{metal}}(z)$ are the electron densities for the isolated molecule and metal subsystem, respectively. In the case of the decorated electrode the metal includes the NH_2 fragment.

The electric field and electrostatic potential associated with charge rearrangement upon adsorption across the molecular junction can be obtained by solving the one-dimensional Poisson equation for the plane-averaged charge density difference:

$$\nabla E(z) = \frac{e\overline{\rho}_{\text{diff}}(z)}{A\epsilon_0}, \tag{2}$$

where A is the area of the simulation cell. From the previous expression $V(z)$ is obtained by integrating the electric field along the z-direction:

$$V(z) = -\int_{-\infty}^{z} E(z')dz'. \tag{3}$$

Figure S2a,b shows the electron density difference upon adsorption of NH_2 and NH_3 respectively. Carbene atoms are described by ghost orbitals. Figure S2c shows the electron density difference averaged over the xy-plane and plotted along the normal to electrode surface (z-direction). While NH_2 has accepted a certain amount of e^-, the NH_3 group behaves as an electron donor.

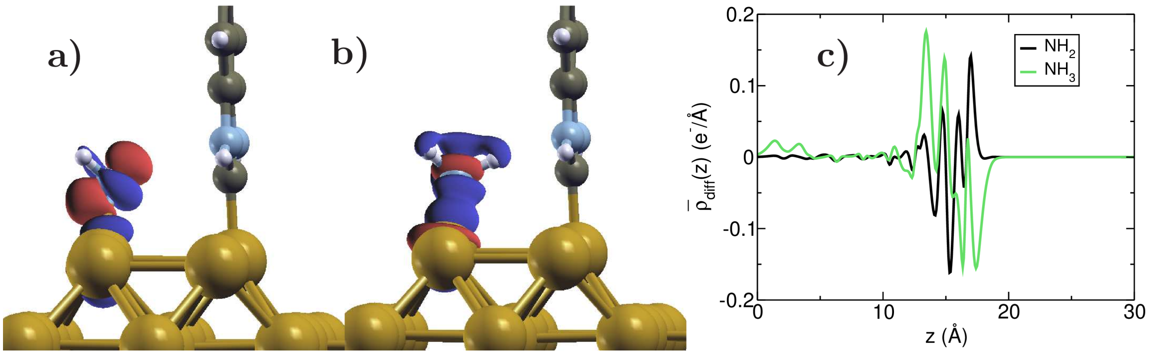

Figure S2: Electron density difference associated to the adsorption of a) NH_2 and b) NH_3. For carbene atoms have been used ghost orbitals. c) Plane-averaged electron density difference along the z-direction (surface normal). While NH_2 has accepted a certain amount of e^-, in the case of the NH_3 the adsorbate has lost e^-.

Characterization of absorbate vibrational modes

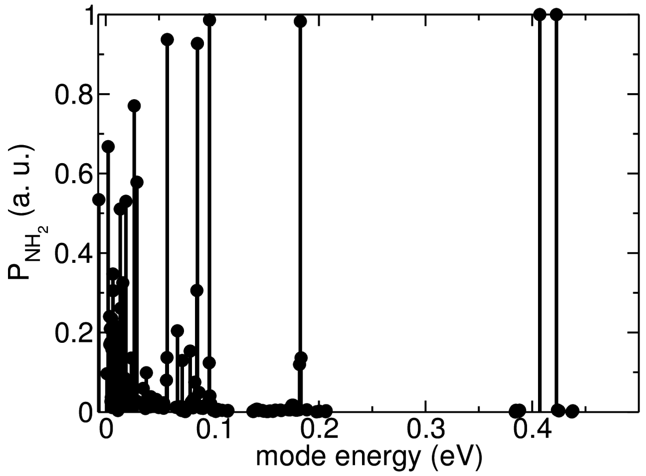

Figure S3: Projection of the eigenvectors of the dynamical matrix over the adsorbate atoms.

Populations of all vibrational modes with a lower damping rate

Figure S4 shows the population and associated stored energy of each vibrational mode as a function of the external bias calculated using a damping rate $J = 4 \times 10^{10}$ Hz. Populations of carbene modes are lower in the presence of the NH_2 adsorbate across the whole bias range considered.

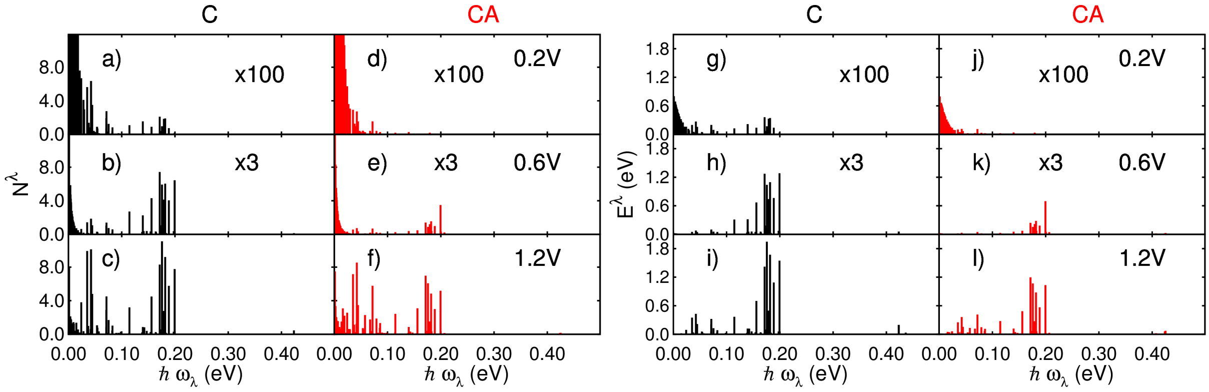

Figure S4: a–f) Populations of all vibrational modes at 0.2, 0.6 and 1.2 V for the junction with and without the NH_2 adsorbate. A damping rate J of 4×10^{10} Hz has been used. g–l) Associated stored energy of all modes for the two geometries considered at 0.2, 0.6 and 1.2 V. In the presence of NH_2 vibron populations and stored energies of NHC modes are in general lower than for the clean junction.

Left-projected spectral function at 1.0 V

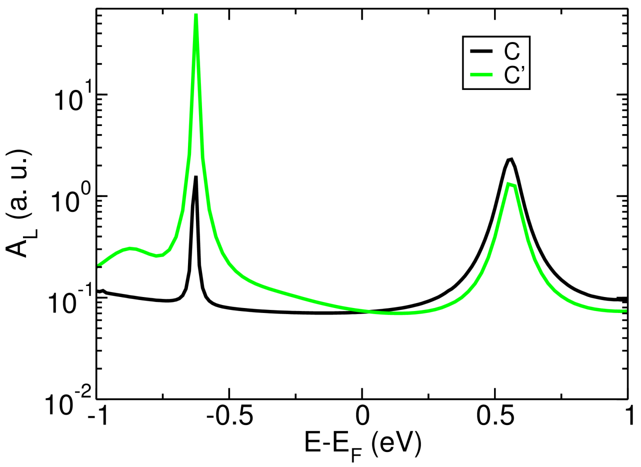

Figure S5: Left spectral function at 1.0 V for the **C** and **C'** structure.

Emission and absorption rates as a function of bias

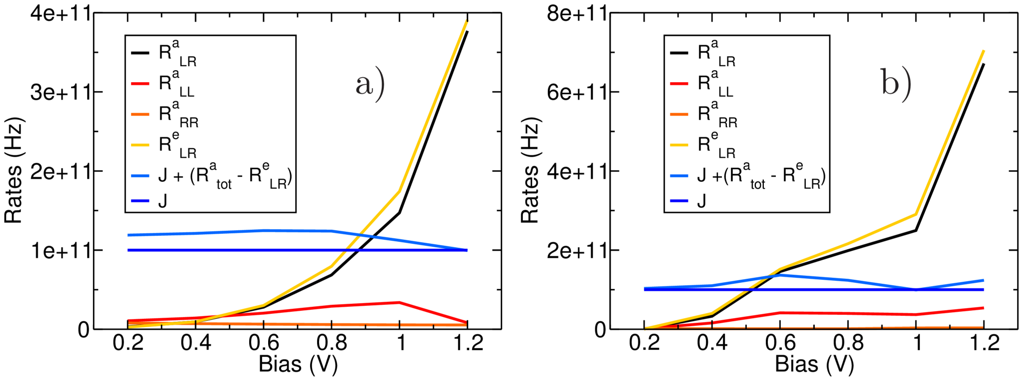

Figure S6: Emission and absorption rates as a function of applied bias for the modes at a) 35 meV and b) 176 meV for the **C** structure.

Carbene vibrational mode at 176 meV

Figure S7 shows the vibrational mode at 176 meV with one of the highest population and stored energy.

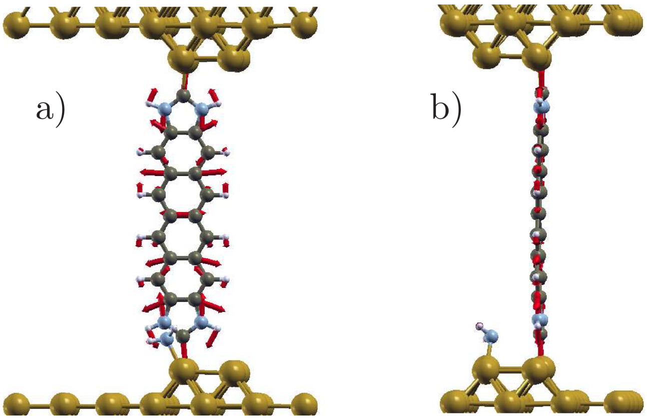

Figure S7: a) Front and b) side view of the vibrational mode at 176 meV.

Change induced by adsorbate states in the populations of all vibrational modes

Figure S8 shows the change induced by adsorbate states in the populations of all vibrational modes as a function of the external bias using a damping parameter $J = 4 \times 10^{10}$ Hz.

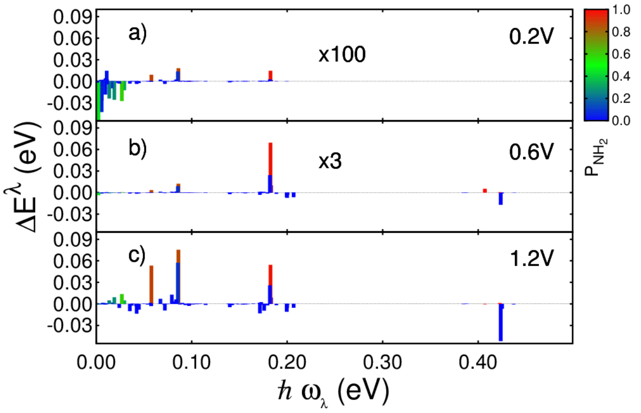

Figure S8: Change in energy stored in each vibrational mode when setting the contributions of the NH_2 group to zero in the spectral functions of the **CA** junction at 0.2, 0.6 and 1.2 V. A damping rate J of 4×10^{10} Hz has been used. The color bar indicates to which extent the vibrational modes are localized on the adsorbate.
